# Multiple Sclerosis Decreases Explicit Counterfactual Processing and Risk Taking in Decision Making

**DOI:** 10.1371/journal.pone.0050718

**Published:** 2012-12-05

**Authors:** Samanta Simioni, Myriam Schluep, Nadège Bault, Giorgio Coricelli, Joerg Kleeberg, Renaud A. Du Pasquier, Markus Gschwind, Patrik Vuilleumier, Jean-Marie Annoni

**Affiliations:** 1 Department of Clinical Neurosciences, University Hospital and University of Lausanne, Lausanne, Switzerland; 2 Institut des Sciences Cognitives, Centre de Neuroscience Cognitive, CNRS, Bron, France; 3 Department of Neuroscience, University Medical Center (CMU), Geneva University, Geneva, Switzerland; 4 Department of Medicine, University of Fribourg, Fribourg, Switzerland; Centre national de la recherche scientifique, France

## Abstract

**Introduction:**

Deficits in decision making (DM) are commonly associated with prefrontal cortical damage, but may occur with multiple sclerosis (MS). There are no data concerning the impact of MS on tasks evaluating DM under explicit risk, where different emotional and cognitive components can be distinguished.

**Methods:**

We assessed 72 relapsing-remitting MS (RRMS) patients with mild to moderate disease and 38 healthy controls in two DM tasks involving risk with explicit rules: (1) The Wheel of Fortune (WOF), which probes the anticipated affects of decisions outcomes on future choices; and (2) The Cambridge Gamble Task (CGT) which measures risk taking. Participants also underwent a neuropsychological and emotional assessment, and skin conductance responses (SCRs) were recorded.

**Results:**

In the WOF, RRMS patients showed deficits in integrating positive counterfactual information (p<0.005) and greater risk aversion (p<0.001). They reported less negative affect than controls (disappointment: p = 0.007; regret: p = 0.01), although their implicit emotional reactions as measured by post-choice SCRs did not differ. In the CGT, RRMS patients differed from controls in quality of DM (p = 0.01) and deliberation time (p = 0.0002), the latter difference being correlated with attention scores. Such changes did not result in overall decreases in performance (total gains).

**Conclusions:**

The quality of DM under risk was modified by MS in both tasks. The reduction in the expression of disappointment coexisted with an increased risk aversion in the WOF and alexithymia features. These concomitant emotional alterations may have implications for better understanding the components of explicit DM and for the clinical support of MS patients.

## Introduction

Multiple sclerosis (MS) is a widespread chronic inflammatory disease of the central nervous system that affects primarily the white matter but is also associated with early cortical demyelination and atrophy [1]. Functional alterations in cognitive and affective functions are commonly observed and play an important role in the everyday disabilities of MS patients. Several studies have demonstrated deficits in frontal lobe functions, including impaired working memory [2], diminished verbal fluency [3], increased sensitivity to interference [3] and impaired conceptual reasoning [4]. Other behavioral studies reported associations between the occurrence of depression and damage in frontal and temporal cortices [5], as well as a lack of functional connectivity linking prefrontal areas to the amygdala during emotional processing [6].

Decision making (DM) is another important function known to implicate prefrontal areas, but is often neglected in clinical neuropsychological assessment and rarely studied in MS patients. A certain number of studies of MS patients ([7], see also below) have focused on DM under ambiguous conditions (i.e., where the risk associated with a choice is not explicitly given) by using the Iowa Gambling Task (IGT) [8]. This test allows a sensitive detection of DM impairments in several neurological conditions and informs on the ability of a subject or a patient to modify its quality of DM, or on its eventual insensibility to monetary losses. To solve the task successfully, subjects have to decipher the relevant but implicit rules based on the feedback received following each choice, as well as to deal with long-term strategies to maximize gains, which can be accomplished by following their subjective affective feelings and hunches as proposed by the Somatic Marker hypothesis [9]. IGT performances and strategies are influenced by both executive [10] and emotional processes [8].

Recent studies using the IGT have shown that DM processes are defective in all MS subtypes, i.e., relapsing-remitting (RR) [11]–[13], as well as primary progressive and secondary progressive MS [14], which is consistent with the impact of white-matter loss on both executive and emotional functions. While DM can be preserved in very early RRMS patients [15], we found by means of a two-year follow-up study that such abilities rapidly declined over time, independently of other disease-evolution markers [7]. All these results underscore the fact that MS patients may fail in choosing advantageously when confronted with ambiguous situations.

The exact mechanisms underlying ambiguous DM deficits in MS are complex, but seem to rely at least partly on affective modifications associated with the disease. MS patients do indeed have difficulties in expressing or describing emotions [16], [17]. We previously found an association of performance in the IGT with lower anxiety scores and decreased emotional experience as measured by skin conductance responses [11], but not with classical executive tests, suggesting specifically impaired affective processes in DM under ambiguity. In fact, affective modifications induced by MS include alexithymia and some difficulties in recognition of facial emotions, and impaired emotional reactivity to negative stimuli is emerging as a consistent feature of patients with MS [18], [19]. By using alternative versions of the IGT, Nagy et al. [13] also showed that DM deficits in MS seemed to mimic those of patients with ventromedial prefrontal cortex (VMPFC) damage, and appeared to be driven by recent outcomes independently from gains or losses, rather than by an overall increased sensitivity to reward and risk taking behavior. Such behavior, driven by short-term benefits, has sometimes been called ‘myopia for the future’ [20].

In daily life, a certain number of situations in which decisions have to be made offer explicit information about the potential consequences of the choice, thus requiring the subjects to decide between alternatives that are defined in terms of probabilities and associated with known rewards and punishments. These kinds of explicit decisions, called “decisions under risk” [21], also seem to implicate cognitive (probabilistic) and emotional processes [22]. Among the various tasks developed to test DM under risk, the Wheels of Fortune task (WOF) is an explicit task in which the subjects see online what they could have won or lost if they had chosen differently. This task has been used to show that DM strategies can be influenced by anticipation of regret [23]. The WOF was developed on the basis of the Decision Affect Theory [24] which emphasizes the role of anticipated affective impacts of decisions on future choices. By confronting the participant with the comparison of “what is” with “what might have been” (counterfactual thinking), this task allows cognitively-generated emotions such as disappointment and regret to be measured [23], [25], [26]. The WOF provides a measure of how decisions and risk taking are modified under the pressure of such emotions.

Another explicit DM task is the Cambridge Gamble Task (CGT), a well known paradigm in which the subject can visualize options while deciding the exact chance of winning or losing his bet, which allows a differentiated assessment of impulsive response tendencies and real risk taking. Moreover, classical executive processes such as working memory are minimized in the CGT, since the information required to make the decision is presented explicitly on each trial. The CGT was developed to assess DM and risk taking [21] in patients with damage to the VMPFC [10], [27], [28]. This task has revealed increased risk preference in these patients, whereas their probabilistic judgment (i.e., quality of DM) was similar to that of the controls. Performance of focal brain-damaged patients on this task suggest that VMPFC lesions lead to selective increases in risk-taking behaviors, while lesions in the insular cortex (IC) lead to difficulties in betting calculations [29], [30].

Given the previous evidence for a modification of both emotional experiences [11] and cognitive competencies [3] related to MS, as well as the potential impact of MS lesion on the integration of these two types of information necessary for optimal DM, it is likely that explicit DM might also be impaired or modified in these patients. However, the absence of lesions limited to the VMPFC, and the frequent occurrence of anxiety disorders in MS patients [31], might predict that the pathological modification in DM might not necessarily involve an increase in explicit risk taking [32], but could instead reduce risk taking strategies. To our knowledge, there are no data concerning the effect of MS on DM competence under risk, and no correlations between explicit DM performance and specific performances of cognitive or affective functioning in such patients. Therefore, we examined here the performances of MS patients in two explicit decision situations. We asked whether MS patients would behave differently from healthy controls, particularly in situations where decisions are based on emotional signals. For this purpose, we used the two classic explicit gambling tasks, the WOF and CGT mentioned above, all given to the same patients.

We predicted that MS patients would show changes in explicit DM for both the WOF and CGT, and this should be associated with modifications in emotional domains in these patients. Our main hypotheses were the following:

1) MS patients will present a different choice behavior of DM under risk than controls; this might be the case in both the WOF and CGT; 2) Such modifications will not necessarily reflect increased risk taking -unlike in patients with restricted lesions to VMPFC- but MS patients might even rather present risk aversion, at least in situations where the anticipated affective impact of decisions can influence future choices (such as the WOF); 3) Such DM modifications in MS patients might be associated with specific emotional changes, as previously observed for implicit DM [11], but not necessarily associated with cognitive changes. Therefore, all patients were carefully examined with a systematic cognitive evaluation and measures –during the WOF- of both explicit and implicit emotional reactions.

## Materials and Methods

### Study Subjects

RRMS patients conforming to the McDonald diagnostic criteria [33], [34] were contacted for the study. Inclusion criteria were: (1) mild to moderate neurological disability, but with unimpaired ambulation (Expanded Disability Status Scale [EDSS] ranging from 1.5 to 3.5) [35]; (2) no clinical relapse and no corticosteroid therapy for at least six weeks before inclusion in the study; (3) no diagnosis of major depression, alcohol or drug abuse or other psychiatric disorders according to the DSM-IV criteria. Seventy-two patients, aged between 18 and 48 years old, were analyzed. Thirty-nine patients were treated with disease-modifying therapies (interferon β-1a or 1b in most cases) for a mean period of 3.3 years (ranging from two months to nine years), ten were taking antidepressants for minor mood symptoms which were not severe enough to fulfill the diagnostic criteria for depression as assessed in a psychiatric interview, and five had had symptomatic treatment for fatigue (amantadine or modafinil). The control group consisted of 38 healthy volunteers matched for age, gender and education, with no history of alcohol or drug abuse, major psychiatric disorders (major depression, psychosis, untreated bipolar disorders), head trauma, other neurological disorders, or systemic illness.

### Standard Protocol Approvals, Registrations and Patient Consents

The study was approved by the local university’s Ethics Committee, and all subjects gave written informed consent for their participation in accordance with the Declaration of Helsinki. The Name of the Ethic Committee is the following: “Commission d’Ethique de la recherche clinique, Faculté de médecine et de biologie, Université de Lausanne, Switzerland”. The name of the accepted project (2007) was: “Dissecting the decisional process in patients with Multiple Sclerosis”. In order to maintain motivation, participants were informed that they would receive money as a function of their final gains in the DM tasks. For ethical reasons, in actual fact they all received 20 Swiss Francs at the end of the study.

### Neuropsychological Examination

All participants underwent a neuropsychological examination, to test whether DM deficits were associated with specific pattern of cognitive deficits. The Brief Repeatable Battery of Neuropsychological Tests (BRB-N) [36], [37] was used to assess verbal memory (Selective Reminding Test [SRT]), spatial memory (10/36 Spatial Recall Test), sustained attention/information processing speed (3-second version of the Paced Auditory Serial Addition Test [PASAT], Symbol Digit Modalities Test [SDMT]), and verbal fluency in semantic cues (Word List Generation [WLG]). In addition to the BRB-N, we administered a task which assessed more complex executive skills (Stockings of Cambridge [SOC] from the Cambridge Neuropsychological Test Automated Battery [CANTAB]) [38].

### Emotional and Behavioral Examination

At the end of the second testing session, subjects filled out questionnaires assessing mood (Hospital Anxiety and Depression scale, HAD) [39] and behavioral changes (Dysexecutive Questionnaire, DEX) [40]. The DEX was used for quantifying behavioral disturbances commonly associated with executive impairment and has already been used in MS studies [41]. This last questionnaire was aimed at investigating whether specific behavioural changes/symptoms in MS are associated with eventual DM changes. The 20 items of the DEX encompass broad areas of likely changes (impulsivity, apathy, desinhibition, intentionality, etc.). Each item is scored on a 5-point scale ranging from “never” to “very often” (0 to 4).

### Wheels of Fortune

**Figure 1:**
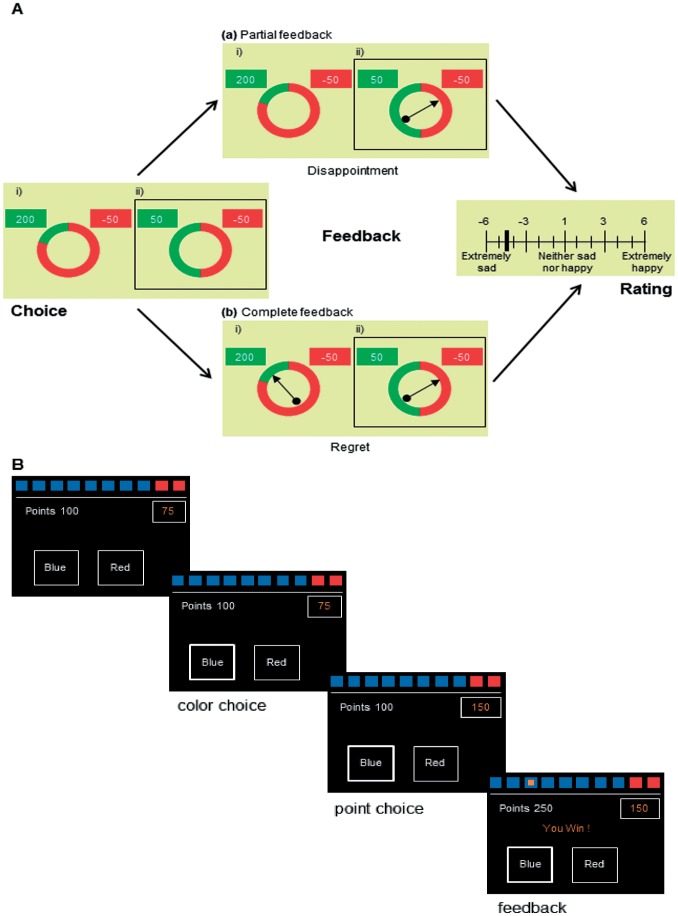
Description of the two gambling tasks. A) Wheel of Fortune (WOF): the subject (with SCR recorded) has to choose between two possible lotteries with different risk and earning possibilities. The subject was asked to choose one of the two wheels by pressing a left or a right button (choice period). A spinning arrow then appeared at the center of the wheel, spins for a variable duration (wait period), and stopped revealing the outcome(s) (feedback period). In the partial feedback condition (30 trials) the subject does not know the outcome of the other lottery; in the complete feedback condition (30 trials) both outcomes are presented. At the end of each trial, subjects had to indicate their affective state using a rating scale. B) Cambridge gambling task (CGT): the subject has to bet points on a choice associated with a given level of risk. Trials are run in blocks (two sets of four blocks), each containing nine trials. A row of ten boxes (red or blue, with a ratio varying across trials) is presented at the top of the screen. Participants are told that a yellow token was hidden in one of the boxes. They then have to guess whether it is in a red or blue box (color). Then they decide how many of their points they wanted to gamble on their choice (point choice) by pressing when they choose: available bets (5, 25, 75, 95 and total) are presented on the right of the screen in a ascending or descending sequence. Then feedback is given about gain or loss and total ongoing fortune (left).

The Wheels of Fortune (WOF) task [23] was used to measure the emotional strategies associated with DM under risk. This task had previously shown that advantageous decisions in healthy controls could be induced through prior experiencing disappointment and regret (Fig. 1A) [23].

Two wheels were presented on a computer screen (Gamble 1 and Gamble 2). Each wheel was divided into two sectors (black and light blue) associated with different amounts of money. The size of each sector represented the probability of obtaining the proposed outcome. The possible outcomes for each individual gamble were visible on the screen and were formed by any pair of the following values: −50, +50, −200, +200 (units corresponding to cents in Swiss francs), and associated with different outcome probabilities (0.8, 0.2, and 0.5). The subject was asked to choose one of the two wheels by pressing a left or a right button (choice period). A spinning arrow then appeared at the center of the wheel, turned for a variable duration (wait period), and stopped in one of the two sectors, revealing the outcome which resulted in a financial gain or loss (feedback period). At the end of each trial, subjects had to indicate their affective state using a rating scale ranging from −50 (extremely sad) to +50 (extremely happy).

The task included two conditions given in separate blocks. In the “partial feedback” condition (PF; 30 trials), the spinning arrow and the related outcome were apparent for the selected wheel only. In this condition, the unfavorable comparison of the obtained outcome with a more favorable counterfactual (i.e., unobtained) outcome may have induced disappointment related to the financial consequence of a decision [23]. By contrast, in the “complete feedback” condition (CF; 30 trials), spinning arrows appeared, rotated and stopped in both the selected and the non-selected wheels, revealing both outcomes to the participants. CF trials induced not only disappointment but also regret, by showing the outcome that would have been obtained if participants had selected the other gamble (counterfactual outcome).

### Skin Conductance Response Recording

Skin conductance responses (SCRs) were recorded during the WOF task using the PowerLab/GSR amplifier system (AD Instruments GmbH, Spechbach, Germany). This was done in order to obtain the physiological correlate of regret, which consisted of an increase of SCRs in the CF condition due to counterfactual processing, as compared to the PF condition of the WOF. The SCRs represent an indicator of sympathetic nervous system activation, which is believed to contribute to the process of making advantageous choices for the organism.

SCR data were acquired continuously using flat-surface electrodes placed on the non-dominant hand and stored for off-line analysis using a second computer running Chart v4.2 software. The SCRs of interest were those generated during the five-second interval following the viewing of the obtained outcome (feedback period, post-choice SCRs). Sixty five-second time points were recorded for each subject. Artefactual signals (e.g., the subject’s movements) were cleared manually. Mean amplitudes (microSiemens, µS) recorded during each five-second time window were analyzed (i.e., the mean of the data points obtained in the five-second selection). Baseline SCR activity was assessed using three measurements per subject recorded: (1) at rest; (2) in response to a loud noise; and (3) after a deep breath. This method had been applied in a previous study [11] and had been shown to be reliable.

### Cambridge Gamble Task

The Cambridge Gamble Task (CGT) [21] was used to measure DM (Fig. 1B). Participants sat in front of a computer touch screen. Trials were run in blocks (two sets of four blocks per subject), each containing nine trials. A block could finish prematurely if it ended in bankruptcy. At the beginning of each block, they were given 100 points. A row of ten boxes (red or blue, with a ratio varying across the trials) was presented at the top of the screen and participants were told that a yellow token was hidden in one of the boxes. They then had to guess whether it was in a red or blue box and to decide how many of their points they wanted to gamble on their choice (5%, 25%, 50%, 75% or 95%, given in a progressively ascending or descending order depending on the blocks). A winning choice was rewarded by the total of points gambled, whereas a losing choice was punished by subtracting that number of points. The probability of each choice being correct was indicated to the subjects by the ratio between red and blue boxes. This produced a variety of situations, ranging from those in which one outcome was the most likely (e.g., nine red boxes to one blue box) to those in which both outcomes were almost equally likely (six to four). We used the standard version provided by CANTAB (http://www.cantab.com/cantab-tests-cambridge-gambling-task.asp) with a 5∶5 ratio of red-blue boxes also included in the design to ensure that participants perceived the task as a random trial sequence. Therefore, it was hypothesized that the CGT allowed participants to apply cognitive strategies, such as probabilistic judgment, in order to decide advantageously. The ascending and descending sequence in which potential wins were proposed enabled us to differentiate patients with impulsive response tendencies from patients with real risk preference (i.e., risk-preferent patients had to wait if they wanted to place high bets in the ascending conditions). Measures of behavior choices were quality of DM (the proportion of trials where participants chose the more likely outcome) and deliberation time.

### Statistical Analyses

Statistical analyses were conducted using a STATA software package (Version 10.0). Non-parametric tests were applied to all the demographic and behavioral data because (1) the CGT and WOF data could not be transformed successfully to reach normality, and (2) the distribution of the WOF emotional ratings is by definition non-normal as the scale is restricted (values available from −50 to +50) and thus cannot be considered as a continuous variable. Differences were examined using Chi2 tests for the comparison of categorical variables and Wilcoxon signed rank tests for the comparison of continuous variables. To measure the possible influences of executive, attentional and emotional variables on decisional performances, we then computed single Spearman correlation analyses with certain outcome measures in the following executive tasks: SOC, SDMT, and PASAT.

For the WOF task, we first analyzed the emotional evaluations of decisional outcomes and then tested two models of choice computed by regression analyses, using a panel logit procedure with an individual random effect. The panel data analysis took each subject as the unit and the trial as the time. The random effects model was used as the default model, and the parameters were estimated by maximum likelihood. This statistical procedure is extensively described elsewhere [23], [26]. The first model integrated the effects of anticipating disappointment (d, negative emotion expressed in the PF condition when facing a negative outcome) and regret (r, negative emotion induced by the counterfactual comparison of a negative obtained outcome with a more advantageous outcome for the rejected alternative) in addition to the maximization of expected values (EV, choices of the most favorable odds, reflecting quality of DM and risk taking). In fact, as we have already mentioned, a subject obtaining an outcome lower than expected might experience disappointment or regret. The greater the difference between the expected and the obtained outcome, the more intense this negative feeling will be. Thus, to avoid future disappointment or regret, the subject might progressively adapt his or her choice behavior by selecting a gamble that minimizes the difference between the lowest and highest outcomes, weighted by the probability of the worst possible outcome. This is what we call anticipation of disappointment and regret.

In the second model [42], we used the logit regression to estimate the probability of the participant choosing the first gamble, as a function of the difference in EV (dEV) and standard deviation (a measure of risk) between the first and second lottery:
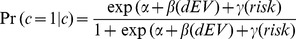



The variables dEV and risk are defined as follows:
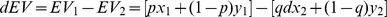


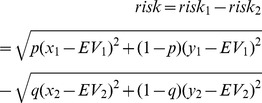
where x_1_, y_1_ and x_2_, y_2_ are the two possible outcomes of the first and the second lotteries respectively, with x_1_>y_1_, and x_2_>y_2_. The probability of x_1_ is p and the probability of y_1_ is (1–p). The probability of x_2_ is q and the probability of y_2_ is (1–q). Thus a positive (negative) and significant dEV or risk coefficient indicates that subjects consistently choose the lottery with the highest (lowest) expected value or level of risk respectively.

For the CGT, choice behavior and betting behavior were analyzed separately [30] on the basis of the scores calculated through the CGT program. It should be noted that trials with a 5∶5 ratio of red-blue boxes, included in the design to mimic a random trial sequence, were excluded from the statistical analyses. The analysis of the participant’s betting behavior was also limited to the trials in which the subjects chose the most likely color, in order to maintain independence from choice behavior. The patients’ and controls’ scores were directly compared using Mann-Whitney non parametric tests.

## Results

### Demographic, Clinical and Neuropsychological Data

The demographic, clinical and neuropsychological characteristics of all the participants are reported in Table 1. RRMS patients and controls did not differ in terms of age, years of education or gender.

**Table 1:** Demographics, clinical neuropsychological characteristics of MS patients and controls.

	Controls (n = 38)	MS patients (n = 72)	p-values
Gender^1^, women	24 (63%)	46 (63%)	0.85
Age, y	32.4±7.6	34.6±6.3	0.11
Education, y	15.4±2.5	14.6±2.8	0.09
Disease duration, y	–	5.06±3.3	–
EDSS score	–	1.9±0.5	–
Immunomodifying therapy^1^	–	39 pt	–
SRT-Long-term retrieval^2^	58.3±12.4	53.3±12.2	0.03
SRT-Delayed recall^2^	11.2±1.6	10.9±1.7	0.38
10/36-Total learning score^2^	23.3±5.1	21.9±5.1	0.27
10/36-Delayed recall^2^	8.6±1.9	8.1±2.1	0.25
SDMT^2^	61.6±9.6	55.0±11.1	0.003
PASAT^2^	32.89±6.8	50.7±9.5	0.055
WLG^2^	30.5±6.2	27.7±578	0.009
SOC-Initial time thinking	0.48±0.6	0.41±1.1	0.47
SOC-Subsequent time thinking	0.3±1.0	−0.7±2.0	0.005
SOC-Problems solved	0.1±0.9	−0.3±1.0	0.5
HAD-A	6.5±3.1	8.5±3.1	0.012
HAD-D	2.32±3.1	3.83±2	0.017
DEX	22.7±8.4	26.2±9.6	0.05

Values are mean ± standard deviation (SD), excepted for ^1^where values are n. ^2^Brief Repeatable Battery of Neuropsychological tests (BRB-N): Selective Reminding Test (SRT), 10/36 Spatial Recall Test (10/36), Symbol Digit Modalities Test (SDMT), Paced Auditory Serial Addition Test (PASAT), Word List Generation (WLG). SOC: Stockings of Cambridge; HAD: Hospital Anxiety and Depression scale.

After correction for multiple comparisons, the RRMS patients scored lower than the controls in measures of attention/processing speed (SDMT: z = −2.97, p = 0.003), and executive functioning (SOC-subsequent thinking time: z = −2.81, p = 0.005). Concerning emotional scores, the RRMS patients reported higher anxiety (z = −2.51, p = 0.012) scores in the HAD.

### Emotional Decision Making: The Wheels of Fortune Task

#### Choice behavior

**Figure 2:**
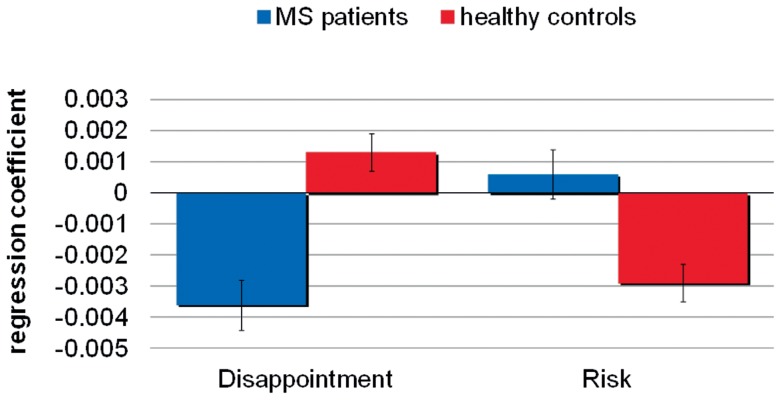
Model of choice integrating the effects of disappointment and risk in addition to the maximization of expected values. Negative coefficients reflect minimization of disappointment or risk. Model A integrates the effects of anticipating disappointment (d) in addition to the maximization of expected values (EV). MS patients did not minimize d. Model B integrates the effects of risk in addition to the maximization of expected values (e). Healthy controls were risk neutral while MS patients were risk averse.

Results based on regression analyses (Table 2A, Fig. 2) showed that the controls, but not MS patients, chose by anticipating disappointment (β = −0.0036, p<0.001). The second regression analysis (Table 2B, Fig. 2) revealed that the RRMS patients were more risk averse (β = −0.0029, p<0.001) relative to the healthy controls, who were risk neutral (β = 0.0013, p = 0.1).

**Table 2:** Choice behavior in the Wheels of Fortune task for MS patients and healthy controls (regression analyses, panel logit with individual random effect).

	Healthy controls (n = 38)				MS patients (n = 72)			
A								
choice	Coeff	Std Err	z	p	Coeff	Std Err	z	p
**EV**	0.0338	0.0018	18.49	<0.001	0.0271	0.0012	22.26	<0.001
**d**	−0.0036	0.0008	−4.32	<0.001	0.0004	0.0006	0.67	.502
**r**	0.0035	0.0006	5.96	<0.001	0.0054	0.0004	12.19	<0.001
**cst**	0.3300	0.0763	4.32	<0.001	0.1954	0.0520	3.75	<0.001
	Log likelihood = −673.57; Wald chi2(2) = 552.77; prob>chi2 = 0.0000				Log likelihood = −1298.23; Wald chi2(2) = 1100.22; prob>chi2 = 0.0000			
**B**								
**choice**	**Coeff**	**Std Err**	**z**	**p**	**Coeff**	**Std Err**	**z**	**p**
**EV**	0.0297	0.0013	23.39	<0.001	0.0284	0.0009	32.33	<0.001
**risk**	0.0013	0.0008	1.57	0.116	−0.0029	0.0006	−4.58	<0.001
**cst**	0.1876	0.0678	2.77	0.006	0.0391	0.0480	0.81	0.415
	Log likelihood = −729.01; Wald chi2(2) = 555.41; prob>chi2 = 0.0000				Log likelihood = −1397.32; Wald chi2(2) = 1109.13; prob>chi2 = 0.0000			

A. Model of choice integrating the effects of anticipating disappointment (d) and regret (r) in addition to the maximization of expected values (EV). Both MS patients and controls chose anticipating r and maximizing EV. MS patients did not choose anticipating d.

B. Model of choice integrating the effects of risk in addition to the maximization of expected values (e). Healthy controls were risk neutral while MS patients were risk averse.

#### Emotional evaluation

**Figure 3:**
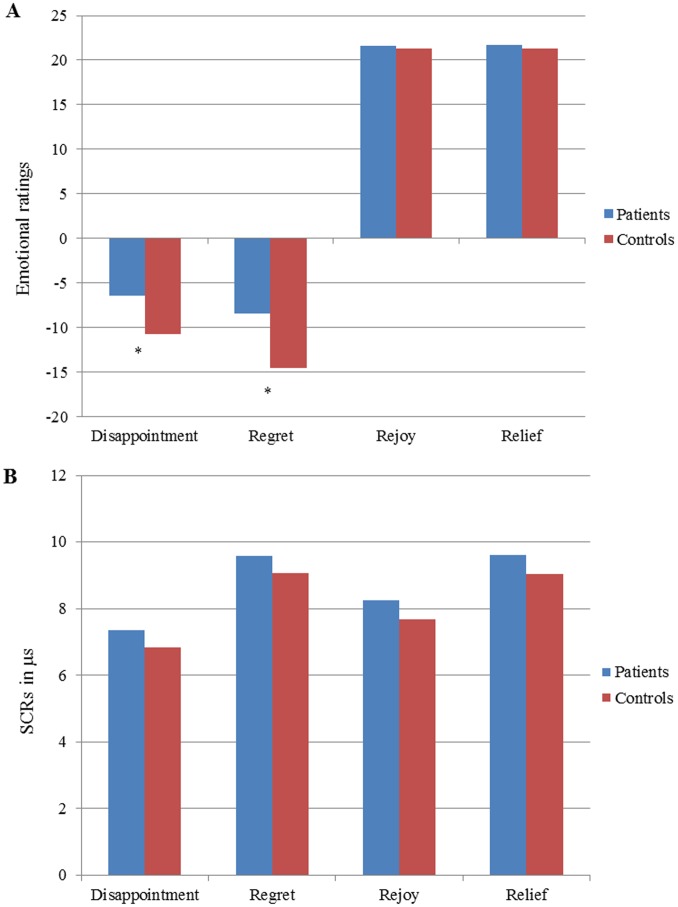
Emotional processing in the WOF task. A. Diminished negative emotional ratings in RRMS patients. Mean emotional ratings were plotted for *disappointment* (comparison of an obtained outcome with a more favorable unobtained outcome in partial feedback condition), *regret* (comparison of an obtained outcome with a more favorable unobtained outcome in complete feedback condition), *rejoy* (comparison of an obtained outcome with a less favorable unobtained outcome in partial feedback condition) and *relief* (comparison of an obtained outcome with a less favorable unobtained outcome in complete feedback condition). Wilcoxon signed rank tests between groups for disappointment and regret: disappointment (z = −2.45, p = 0.01) and regret (z = −2.38, p = 0.02). B. Comparable emotional arousal in SCRs for RRMS patients and controls. Mean SCRs during feedback plotted for disappointment, regret, rejoy and relief. No statistical differences between groups.

Results from the emotional ratings in the WOF showed that the RRMS patients reported less negative affective reactions, as compared with the controls, for both disappointment (z = −2.45, p = 0.01) and regret (z = −2.38, p = 0.02). In contrast, their explicit expression of positive emotion was comparable (Figure 3A). To test the hypothesis of a general deficit in evaluating negative feedbacks, a linear regression was run separately for positive and negative outcomes testing the influence of obtained outcome, counterfactual outcome (i.e., unobtained by a chosen gamble during the PF and obtained by an unchosen gamble during the CF), and group on the affective ratings. We found no effect of the [obtained outcome x group] interaction for positive outcomes (β = 0.01, p = 0.2), meaning that both groups rated positive outcomes in the same way. By contrast, the effect of the [obtained outcome x group] interaction showed a strong trend after Bonferroni correction towards negative outcomes (β = 0.04, p<0.005), confirming that RRMS patients rated negative outcomes less negatively than the controls. Finally, concerning counterfactual outcomes, there was an effect of counterfactual outcome x group for both the positive (β = 0.01, p<0.005) and negative (β = 0.01, p<0.05) conditions, indicating a general deficit in the integration of counterfactual information in the explicit evaluation process for the RRMS patients. There were no significant correlation between measure of regret and cognitive scores.

#### Skin conductance response recording

SCR baseline reactivity was comparable in RRMS patients and controls at rest (z = −1.46, p = 0.1), in response to loud noise (z = −1.43, p = 0.1) and after deep breaths (z = −1.18, p = 0.2). These baseline effects rule out any global dysfunction of the autonomic nervous system in the patients. During the WOF task, SCRs were significantly increased in both the RRMS patients and controls in response to the presentation of the outcome of the rejected alternative, reflecting the emotional nature of this information (RRMS patients: z = 2.90, p = 0.004; controls: z = 2.09, p = 0.04). This preserved effect in RRMS also reveals an implicit integration of counterfactual information that differed from the lack of explicit use in their affective ratings. In contrast with emotional ratings reported by participants after each trial, SCRs reactivity did not differ between the RRMS patients and controls, neither for positive (z = −0.63, p = 0.50) nor for negative outcomes (z = −0.71, p = 0.50) (Figure 3B).

### Cognitive Decision Making: the Cambridge Gamble Task

#### Choice behavior

**Figure 4:**
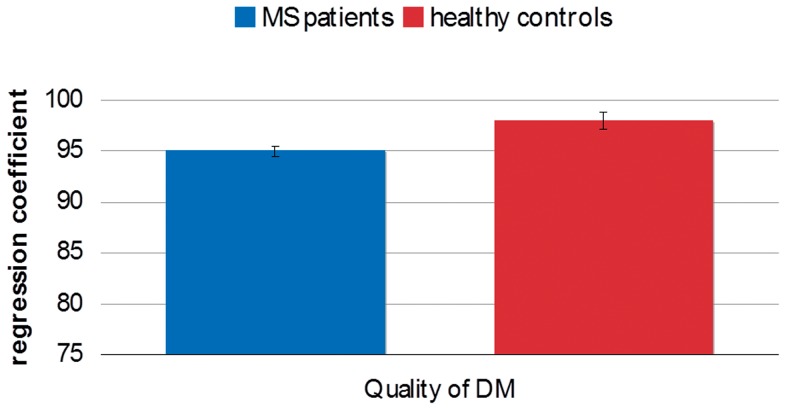
Quality of DM in the Cambridge Gambling Task. Comparisons in terms of choice behavior between MS patients and controls in the CGT (% of best choices).

The RRMS patients differed from the controls (Table 3, Fig. 4) for both measures of choice behavior: they selected the most favorable odds less often (95% ±0.5 of trials vs. 98% ±0.8 for controls; z = 2.44, p = 0.01) and exhibited longer deliberation times before choosing (2413±905 ms vs. 1934±541 ms for controls; z = −3.38, p<0.001). However, when computing the total number of blocks ending up in bankruptcy (i.e. blocks interrupted when the total score reached ≤1 point), no difference between groups was observed (z = −1.32, p = 0.2).

**Table 3:** Comparisons in terms of choice behavior and betting behavior between MS patients and controls in the CGT.

	Controls (n = 38)	MS patients (n = 72)	p-values (Wilcoxon)
**Choice behavior**			
Quality of decision making	98±0.5	95±0.8	z = 2.44, p = 0.01*
Deliberation time (ms)	1934±541	2413±905	z = −3.34, p = 0.005*
**Betting behavior**			
Risk taking	0.60±0.09	0.58±0.1	z = −1.16, p = 0.24
Risk adjustment	1.6±0.7	1.4±0.9	z = −1.1, p = 0.28

Msec = milliseconds. Values are means ± standard deviation (SD). P-values reflect non parametric comparisons.

* reflects significant difference. After correction for type 1 error, alpha was set at.025 for choice behavior (2 Items) and for betting behavior (idem).

In the MS group, several correlation analyses were computed between the quality of DM and relevant executive performance (three outcome measures of the SOC and performances in the WLG task), attention scores (SDMT, PASAT), affective state (values of the HAD), and behavioral changes (DEX questionnaire). Following Type 1 error correction, Spearman correlation test showed that, in RRMS patients, the quality of DM showed no direct relations with measures of executive functioning. By contrast, deliberation times correlated negatively with attention/processing speed measures (PASAT: r = −0.364, p = 0.002).

## Discussion

The present study is, to our knowledge, the first to assess DM in RRMS patients using two explicit tasks with known outcome probabilities, and a differential recruitment of cognitive and emotional components. Our main hypothesis was that RRMS patients would show modified strategies in explicit DM. Moreover, we looked for decreased risk taking, and modifications in emotional experience. Our results support these hypotheses: (1) RRMS patients showed, in comparison to controls, a poorer quality of DM, as indicated by the fact that they did not maximize the expected values in the WOF task, and that they selected less often the most likely odds in the CGT; (2) RRMS showed greater risk aversion; (3) RRMS patients were less sensitive to counterfactual information in the WOF task and exhibited a decreased sensitivity to explicit negative emotional experience (measured by questionnaire), but not in covert emotional reactions (measured by SCR). On the contrary, a significant association with cognitive functions (but not emotional indices) was found for the CGT task, with a selective correlation between initial decision time and performance in an attentional task (PASAT), suggesting that overall cognitive changes had little impact on choice behavior. These modifications in MS patients are significant although of smaller magnitude in comparison with those reported after orbitofrontal [23] and insular lesions [30], or in comparison with changes detected in DM under ambiguity [11]. In addition, they did not cause increased bankruptcy in DM or overall gain decrease. This suggests that MS modifies, rather than totally disrupts, explicit DM abilities, at least in the mild to moderate phase of the disease.

These behavioral results converge nevertheless with those obtained in studies dealing with DM under ambiguity in similar patients. The latter studies used the IGT and reported a consistent failure of the decisional process within a learning context of simple feedback contingencies [7], [11], [13]. Moreover, the finding of Nagy et al. [13], i.e., that DM impairment in MS is not necessarily associated with increased sensitivity to reward and increased risk taking, nicely dovetail with our data, which suggest greater risk aversion. The role of a decreased emotional reactivity related to changes in DM was hypothesized based on reduced SCRs [11]. In the current study, we now show that RRMS patients may modify their strategies in DM under explicit risk conditions.

### Associated Mechanisms

The correlation of DM mechanisms with overall cognitive and behavioral (DEX) functioning was weak. The only significant association with cognitive performances was found between the CGT deliberation time task and a measure of attentional/working memory performance (PASAT). This correlation may reflect the known slower processing speed in the MS group, and explains also why deliberation time at the CGT is prolonged in MS patients.

Concerning emotional processing, we found a significant relation between counterfactual outcome and group in the WOF, suggesting that a global deficit by MS patients in integrating counterfactual information in the evaluation process may play a direct role in the modification of DM. Different studies have provided behavioral evidence for a role of counterfactual anticipation of regret and relief in DM, but also in probabilistic learning [43]. This raises the question of whether probabilistic learning might be more globally decreased in MS patients and thus might account for this difficulty. To our knowledge, this question, i.e., how patients learn the contingencies between environmental stimuli and their consequences, has been addressed only infrequently in neurological diseases [44]. On the other hand, the deficits in the WOF task in our patients were associated with a failure to rate and use emotional experiences adequately in order to maximize expected values. These results suggest that, like in the IGT [11], modifications in emotional experience may be associated with changes in DM under explicit risk. Moreover, they point to the role of an overt emotional evaluation rather than a somatic implicit marker.

### Neuroanatomical Hypotheses

The correlation between lesion load and cognitive functions was overall weak in MS and we did not perform a systematic quantitative lesion analysis in our study [45], [46]. However, our RRMS patients showed a decrease in betting behavior but with an increase in risk aversion during both DM tasks, suggesting that changes in betting was not due to high risk biases like in VMPFC patients. This result adds further weight to the finding of Nagy et al. [13] that DM impairment in MS patients is not related to an increased sensitivity to reward. In addition, this pattern suggests dissociation between the mechanisms promoting risky choices and risk aversion, which may be differentially affected by focal damage to the VMPFC and by more diffuse white-matter lesions, respectively.

### Risk Aversion and Emotional Reactivity

An important result which was found in both gambling tasks concerned risk aversion, particularly present in the WOF task. It has been proposed that, in cases of impaired DM or probabilistic judgment, the reduction of risk might reflect a compensatory mechanism designed to maintain the final performance [47]. This could potentially provide an explanation for our observations. RRMS patients might behave in a more conservative manner because of a loss of confidence in their choices. A further possible explanation for risk aversion might stem from our findings in the WOF task, where the RRMS patients demonstrated a distinctive alteration of emotional processing. When confronted with negative outcomes during the task, the patients expressed significantly less disappointment and less regret than the controls, whereas the rating of positive emotions was comparable in both groups. This behavior echoes other similar findings in emotional tasks in MS patients [19], and cannot be due to a general disinterest in the task, since this would rather lead to an overall reduction of magnitude of emotional rating. The amplification effect (i.e., the difference in negative ratings obtained in PF vs. CF conditions) which is an indication of regret was also non-significant in these patients, indicating decreased counterfactual processing and deficient anticipation of the negative emotions of losses. In more general terms, these data suggest a deficit in incorporating counterfactual information into the explicit emotional evaluation process. This could reflect a deficit in consciously integrating different sources of information during DM, perhaps due to disconnection lesions in MS patients, and demonstrates that a lack of explicit counterfactual regret may arise without damage to the orbitofrontal cortex (OFC) itself (see [23]).

Strikingly, although RRMS patients reported disappointment and regret to a significantly lesser extent as compared to healthy participants, we could not demonstrate any differences in SCRs between the RRMS patients and healthy controls. Although SCR signals are much more variable than self-report scales, and may therefore be too noisy to demonstrate reliable differences between these groups, our finding contrasts with previous results that suggested that a decreased emotional reactivity may underlie impaired DM in ambiguous situations [11]. Although the role for differences between the tasks used across studies cannot be excluded, one explanation for this difference in SCRs values might be found in the population that was investigated in the current study. Indeed, in this present case, we enrolled only RRMS patients with low neurological disability. By contrast, Kleeberg et al. [11] studied both RRMS and secondary progressive MS patients who had more severe neurological disability (EDSS between 1.5 and 6.5) and longer disease duration (mean 103 months). It might thus be hypothesized that emotional reactivity declines over time as a result of disease progression but is still preserved in early RRMS.

In our view, the lack of self-reported disappointment and regret could reflect a deficit akin to alexithymia. The term alexithymia was first defined by Sifneos [48] to describe an inability to find appropriate words to identify, express or describe emotions, and a difficulty in differentiating feelings from bodily sensations. It has been reported in a variety of neurological diseases [49]–[51]. In MS, alexithymia has also been reported in a large proportion of patients, ranging from 13% to 43% according to various studies [16], [17]. Our work reinforces these clinical observations by providing more experimental perspective on this phenomenon, and by showing for the first time that reduced use of emotional signals or “alexithymia-like” mechanisms might underlie impairments in DM behavior. Furthermore, it has been shown that explicit emotional evaluation (i.e., emotional “appraisal”) may also contribute to effective DM and that emotion regulation strategies (in particular cognitive reappraisal) may have beneficial effects on DM both under risk and under uncertainty [52]. Thus, it is plausible that some form or components of alexithymia might be directly related to increased risk aversion and decreased DM, as observed in our MS patients.

Finally, by showing that alexithymic losses may arise concomitantly to impaired DM performance in RRMS patients, our results highlight that affective processes might contribute to normal DM and its impairment in MS. From a neurobiological point of view, recent neuroimaging studies have emphasized the implication of specific brain areas in alexithymia phenomena, including the anterior cingulate cortex (ACC), the premotor cortex and the amygdala [53], [54]. Therefore, we speculate that the white-matter prefrontal lesion load caused by MS [55] might lead to a decreased structural and functional connectivity within a wide brain network, composed of the OFC, DLPFC, and limbic areas, which might in turn contribute to the impaired integration of cognitive and affective signals, and thus deficient decisional processes in MS. To support this hypothesis at least in part, a lack of functional connectivity between prefrontal areas and the amygdala during emotional processing has been recently reported in patients with early MS in a functional magnetic resonance imaging study [6].

In conclusion, DM under risk was found to be modified in MS patients. However, unlike frontal patients, RRMS patients exhibited an increased risk aversion during the decisional process which might represent a conservative bias due to reduced confidence in their choices. Moreover, impairment of explicit emotional processes (as illustrated by the presence of alexithymia and decreased counterfactual thinking) was also associated with this deficit. DM is an important factor for active patients with MS, for example when deciding for professional, private life, or therapies. Informing them that the disease may have an influence on management of counterfactual information and risk may help them in certain crucial situation. These findings may help not only to better understand the neural bases of DM and their disorders, but also help better listen to and support these (typically young) patients.
